# The Effect of Substrate Biasing during DC Magnetron Sputtering on the Quality of VO_2_ Thin Films and Their Insulator–Metal Transition Behavior

**DOI:** 10.3390/ma12132160

**Published:** 2019-07-05

**Authors:** Chunzi Zhang, Ozan Gunes, Yuanshi Li, Xiaoyu Cui, Masoud Mohammadtaheri, Shi-Jie Wen, Rick Wong, Qiaoqin Yang, Safa Kasap

**Affiliations:** 1 Department of Electrical and Computer Engineering, University of Saskatchewan, 57 Campus Drive, Saskatoon, SK S7N 5A9, Canada; 2 Department of Mechanical Engineering, University of Saskatchewan, 57 Campus Drive, Saskatoon, SK S7N 5A9, Canada; 3 Canadian Light Source Inc., 44 Innovation Boulevard, Saskatoon, SK S7N 2V3, Canada; 4 Cisco Systems Inc., San Jose, CA 95134, USA

**Keywords:** VO_2_ thin films, substrate biasing, transition temperature, sputtering

## Abstract

In this work, VO_2_ thin films were deposited on Si wafers (onto (100) surface) by DC magnetron sputtering under different cathode bias voltages. The effects of substrate biasing on the structural and optical properties were investigated. The results show that the metal–insulator transition (MIT) temperature of VO_2_ thin films can be increased up to 14 K by applying a cathode bias voltage, compared to deposition conditions without any bias. The decrease in the transition efficiency and increase in the transition temperature are attributed to the enlarged grain size, increased defects, and the residual stress in the VO_2_ thin films induced by biasing. The optical transmittance measurements for different thickness films indicate an attenuation coefficient of 3.1 × 10^7^ m^−1^ at 2000 nm or an extinction coefficient of 4.9 in the metal phase. The optical transmittance vs wavelength characteristics point to an indirect bandgap of 0.6 ± 0.5 eV and significant scattering in the bulk and/or at the interface.

## 1. Introduction

Vanadium dioxide (VO_2_) has been widely studied in recent years because of its ability to undergo a reversible metal–insulator transition (MIT) at around 68 °C, from a low-temperature insulating M-phase (monoclinic) to a high-temperature metallic R phase (rutile) [1]. This reversible transition makes VO_2_ an excellent material for ultrafast optical switches, smart windows, Mott transistors, strain and gas sensors, actuators, and so forth [2,3,4,5]. The bandgap of VO_2_ epitaxial films at room temperature have been reported to be about 0.7 eV [6], and would normally classify VO_2_ as a semiconductor rather than an insulator, but the term “insulator’ is commonly used to represent the latter semiconducting phase.

The properties of VO_2_ thin films are strongly dependent on deposition processing parameters. The phase inhomogeneity inVO_2_ bulk or thin films limits their potential applications by rendering the MIT phase transition broad and diffusive [3,7]. The effects of strain, grain size, stoichiometry, substrate temperature, and substrate material have been extensively studied and optimized to achieve good crystallinity with a higher optical transmittance and a higher near-IR switching contrast [8,9,10,11,12,13,14,15]. In addition to optical transmittance and switching contrast enhancement, extensive efforts have been devoted to modifying the transition temperature (*T*_t_) of VO_2_. It was reported that doping and strain control are common methods for *T*_t_ modification [16,17,18,19,20,21,22]. Doping elements of W, Mo, F usually decrease the *T*_t_ [16,17,18,19], while doping elements of Al, Cr, Ti can increase the transition temperature [20,21]. The progress on *T*_t_ reduction has been successful and the *T*_t_ can be brought down by 20 °C [16]. In contrast, the studies on increasing the transition temperature of VO_2_ are not many. Nevertheless, the upper bound of working temperatures for most of the electrical devices utilizing optical switches, transistors, sensors, and actuators are possibly higher than 68 °C due to the accumulated heat during service, which would result in a premature MIT of VO_2_. So, a higher transition temperature of VO_2_ is desirable for these applications.

In previous works, it has been found that the substrate biasing during deposition can change both the microstructure and properties of VO_2_ thin films [23,24,25]. In this work, the effects of substrate biasing are systematically investigated with the goal of increasing the transition temperature of VO_2_. We have considered only single crystal silicon substrates with the view that these VO_2_ films would have to be integrated with today’s silicon-based microelectronics towards hybrid optoelectronic devices. Results reported in this work show that the substrate biasing during the deposition of VO_2_ thin films by DC magnetron sputtering can be effective in increasing the transition temperature. 

## 2. Materials and Methods

Si (100) wafer substrates were ultrasonically cleaned in ethanol and dried in air for use in deposition. VO_2_ thin films were deposited by DC magnetron sputtering onto Si substrates with substrate biasing (i.e., negative bias is applied to the substrate). A high-purity vanadium target (99.95%) was used and sputtered in an Argon (100 sccm) and oxygen (1.3 sccm) atmosphere at a constant pressure of 1.33 Pa and a substrate temperature of 650 °C, respectively. The applied biasing voltage ranged from 89 V to 173 V. The duration of the deposition process was 2 h with a sputtering power of 100 W.

The as-prepared VO_2_ thin films were then characterized using scanning electron microscopy (SEM), Raman spectroscopy, and X-ray Photoelectron Spectroscopy (XPS). The Raman spectra were taken at room temperature using a Renishaw Invia Reflex Raman Microscope (Renishaw, Mississauga, ON, Canada). The operating laser wavelength was 514.5 nm. The optical transmission was measured in the wavelength range of 350–2500 nm using a spectrometer. The transmission measurements were carried out at 300 K (room temperature) and 368 K (above the MIT temperature). The thermal hysteresis loops were obtained by collecting transmittance values versus temperature (with 5 °C intervals both in the heating and the cooling process) at the wavelength of 2500 nm, corresponding to the largest contract in transmittance. The XPS study was carried out using a synchrotron X-ray radiation source at the Canadian Light Source (Saskatoon, SK, Canada). The X-ray photon energy range used for this study was 5.5–250 eV and the spectra were collected using a Photoemission Scienta 100 analyzer (Scienta Omicron, Uppsala, Sweden) under ultrahigh vacuum (10^−10^ torr). The thickness of the as-deposited VO_2_ thin films was measured by a Zygo Optical Profilometer (Zygo, Middlefield, OH, USA) and the grain size was determined on the SEM by using an image processing software SPIP (Version 5.1, Image Metrology, Copenhagen, Denmark).

## 3. Results and Discussion

### 3.1. Microstructure Characterization by Raman

**Figure 1:**
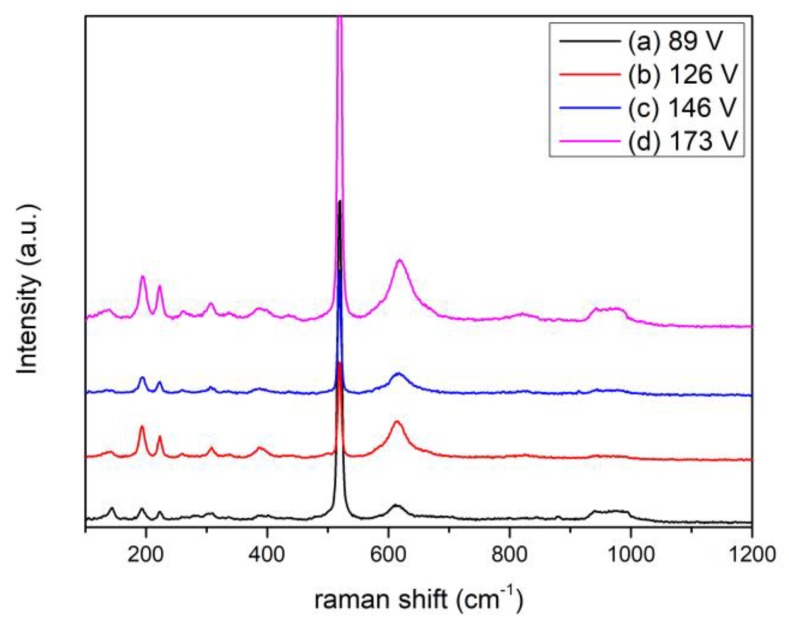
Raman spectra of the as-deposited VO_2_ thin films with substrate biasing: (**a**) 89 V; (**b**) 126 V; (**c**) 146 V; (**d**) 173 V.

In order to study the effect of substrate biasing, we have used single-phase VO_2_ thin films deposited under different substrate bias voltages. This is because nonstoichiometric VO*_x_* thin films usually have significantly different properties compared with single-phase VO_2_ and therefore cannot be used in this work. In our previous work, Raman spectroscopy results have shown to be very useful in studying the stoichiometry of VO_2_ thin films [26]. Therefore, Raman measurements were again used in this study. The deposition conditions are listed in Table 1. Figure 1 shows the Raman spectra of the as-deposited VO_2_ thin films. All the films show typical VO_2_ with the bias voltage ranging from 89 V to 173 V, suggesting that the films have good stoichiometry [26]. The sharp Raman peak at 520 cm^−1^ and the broad peak at around 950 cm^−1^ are from the vibrations of the Si substrate [27].

### 3.2. Surface Morphology Characterization by SEM

**Figure 2:**
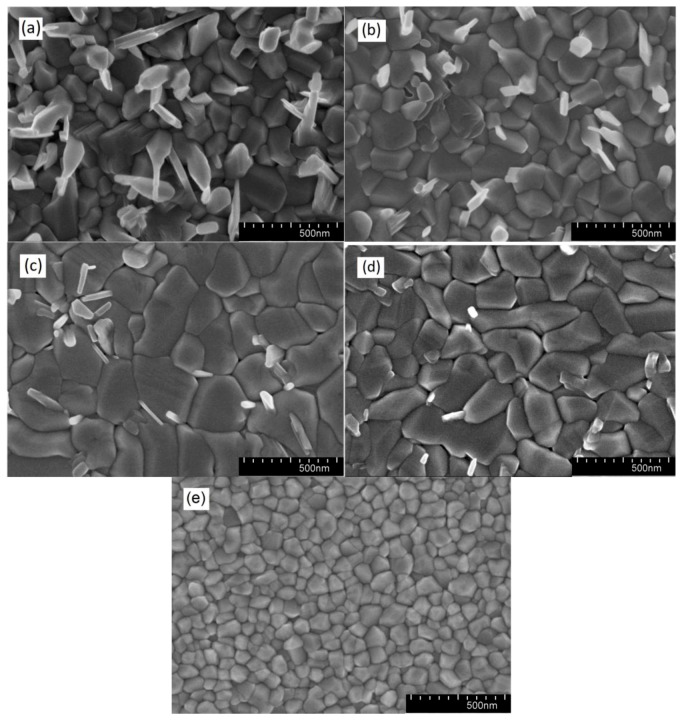
SEM images of VO_2_ thin films deposited on Si substrates for 2 h with bias voltage of: (**a**) 89 V; (**b**) 126 V; (**c**) 146 V; (**d**) 173 V; (**e**) no bias on a sample prepared as in [23].

The surface morphology of the as-deposited VO_2_ thin films was characterized by SEM and the results are shown in Figure 2. All the samples show a polycrystalline structure with a few small grains on top of large grains due to secondary nucleation. In our previous work [23], the average grain size of the VO_2_ thin films deposited for 2 h (150 nm thick) without substrate biasing was 55.4 nm as shown in Figure 2e. However, the grain size of the VO_2_ thin films deposited under the same conditions but with substrate biasing for 2 h (68 nm thick) was much higher and reached 88.6 nm, as shown in Table 1. Moreover, it can be seen that the deposition rate decreased with increase of substrate bias voltage, which is associated with enhanced ion bombardment. Even with reduced deposition rate and thickness, the grain size was significantly larger. This is due to the competing effects of thickness and substrate biasing. Generally, grain growth increases with increasing film thickness since grains can grow further. However, the substrate bias during deposition increases the kinetic energy of the particles impacting the coating; as a consequence, there is an increase in the surface mobility of the atoms forming the film, leading to larger crystalline grains even in thinner films. These results show that substrate biasing is very effective in grain size enlargement. 

### 3.3. Chemical States Characterization by XPS

**Figure 3:**
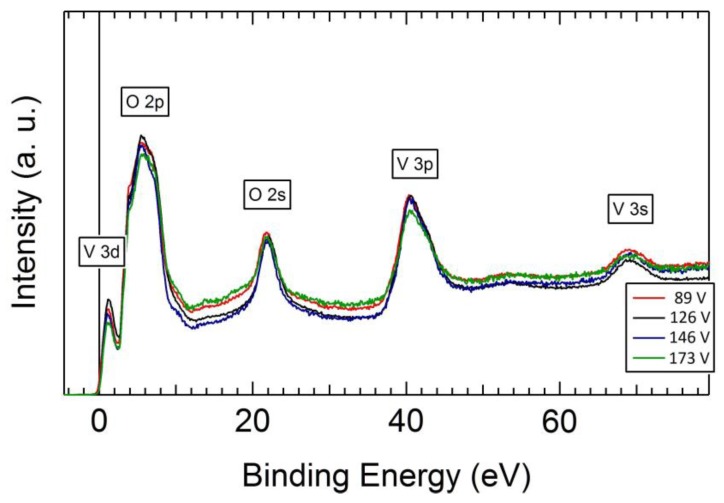
XPS spectra of the as-deposited VO_2_ thin films with substrate biasing.

To fully understand the chemical states near the surface area, we have also taken x-ray photoemission spectroscopy on our as-deposited VO_2_ thin films as shown in Figure 3. The measurement was performed at PGM beamline at the Canadian Light Source, with a beam size of 200 × 200 μm^2^ at room temperature. We can compare the electron states of V 3d and 3p and O 2s in the films deposited under different biasing voltage in Figure 3. It should be mentioned that due to the sensitivity of this technique and the short escape depth of the electrons, the electrons accounting for the spectra are coming from the very surface, at a depth of a few Angstroms from the surface. Therefore, the spectra give information on how the V–V and V–O bonds can be distinguished from each other on the surface. Based on the XPS spectra shown in Figure 3, with all the electronic states considered, it can be concluded that the substrate biasing voltage has little influence on the V–O and V–V bonds at the surface of the thin films. 

### 3.4. Optical Measurements

**Figure 4:**
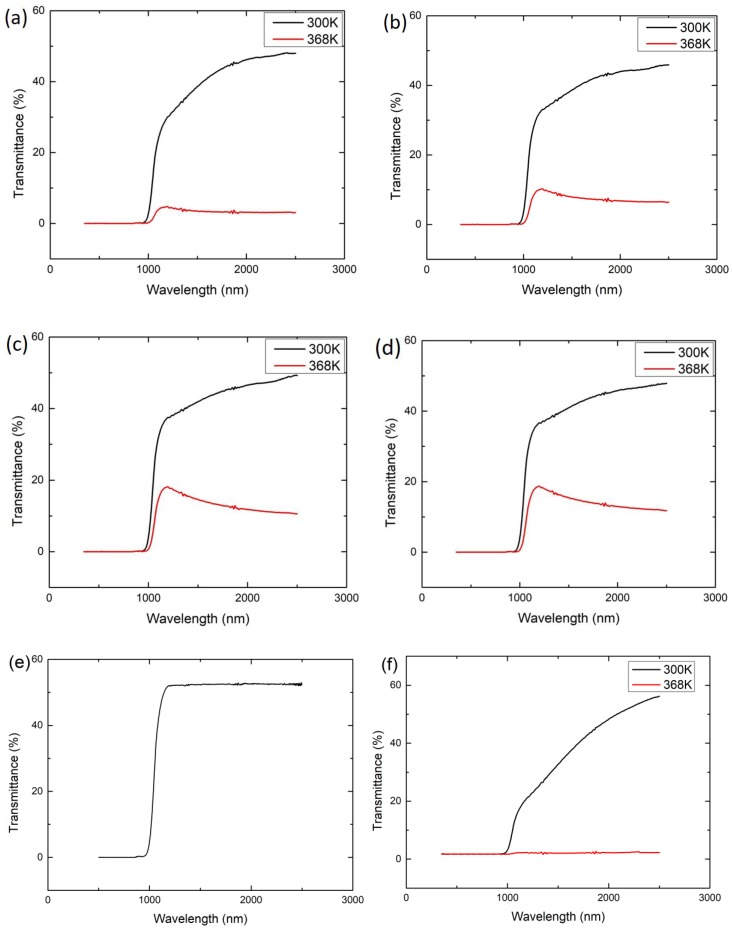
Optical transmittance of the as-deposited VO_2_ thin films with a substrate bias voltage of: (**a**) 89 V; (**b**) 126 V; (**c**) 146 V; (**d**) 173 V; (**e**) bare Si substrate; (**f**) no bias (data extracted from reference [23] and replotted).

**Figure 5:**
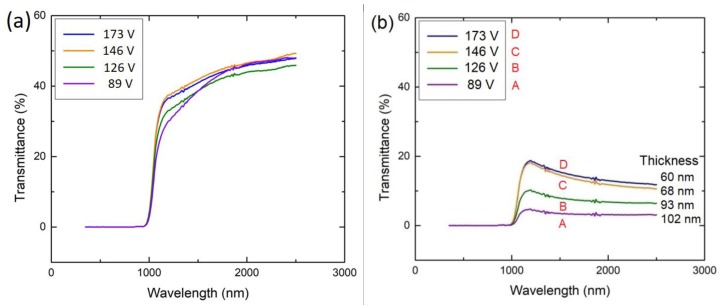
Optical transmittance of VO_2_ thin films deposited under different bias voltage at (**a**) 300 K and (**b**) 368 K.

**Figure 6:**
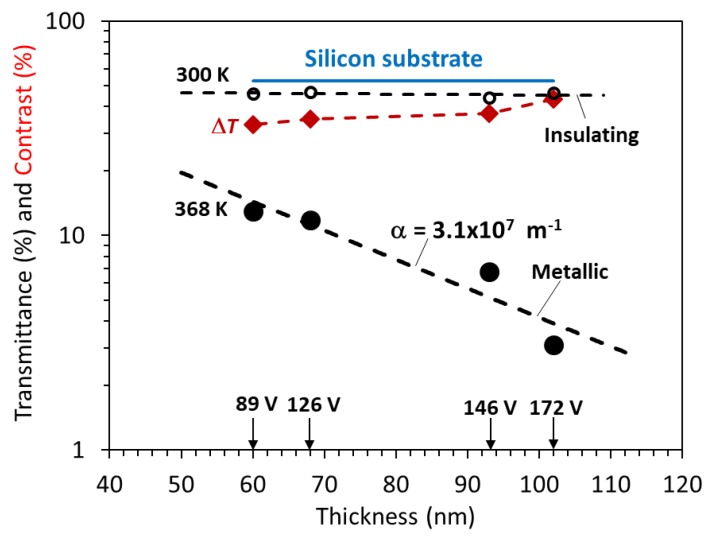
Optical transmittance at a wavelength of 2000 nm in the insulator and semiconductor phases (300 and 368 K, respectively) and contrast Δ*T* vs thickness on a semilogarithmic plot. The dashed lines are exponential fits. The attenuation coefficient of the metal phase *α* ≈ 3.1 × 10^7^ m^−1^.

The transmission spectra of the as-deposited VO_2_ thin films under different biasing conditions are shown in Figure 4. It should be noted that at wavelengths less than 1100 nm (corresponding to the 1.1 eV bandgap of Si), the Si substrate absorbs all the light as shown in Figure 4e, so there is no transmittance. The maximum transmittance reaches about 50% at a wavelength of 2500 nm. The optical transmittance of VO_2_ thin films deposited under different bias voltage at (a) 300 K and (b) 368 K are shown in Figure 5. While in the insulating phase (at 300 K), the transmittance values are very close, in the metal phase (at 368 K), there is distinct dependence on the bias voltage during deposition. However, the actual effect arises from the difference in the thicknesses. The optical transmittance of a homogeneous medium generally decreases with increasing film thickness as *T* = *T*_0_exp(−*αL*), where *α* is the attenuation coefficient, *L* is the film thickness, and the term *T*_0_ is a pre-exponential constant (*T* as *L* → 0). Figure 6 shows the semilogarithmic plots of the optical transmittance (*T* as %) vs the film thickness in the insulating and metal phases. The transmittance contrast Δ*T* (transmittance difference between normal and switched states) vs film thickness is also shown. First, consider the metal phase at 368 K, and notice the clear reduction in the transmittance as the film thickness increases, following an exponential behavior with an attenuation coefficient *α* ≈ 3.1 × 10^7^ m^−1^ at a wavelength of 2000 nm. The reason we can deduce an attenuation coefficient from the ln(*T*) vs *L* plot is that the light intensity is extinguished so much as it passes through the film in this metallic phase that interference phenomena arising from Fresnel reflections are insignificant. This is not the case in the insulating phase. 

The transmittance of the VO_2_/Si structure in the insulating phase at 300 K shows only a very small decrease with increasing film thickness as apparent in Figure 6. The change in *T* with wavelength and thickness in thin films invariably involves interference in the presence of attenuation, which is analyzed below. It should be emphasized that attenuation at 2000 nm in this phase -includes some possible band-to-band absorption and losses arises from scattering within the film as well as from any interface roughness, as discussed below.

The transmittance contrast Δ*T*, an important metric for device applications, increases with film thickness because of the steep decline in the transmission through the metal state.

**Figure 7:**
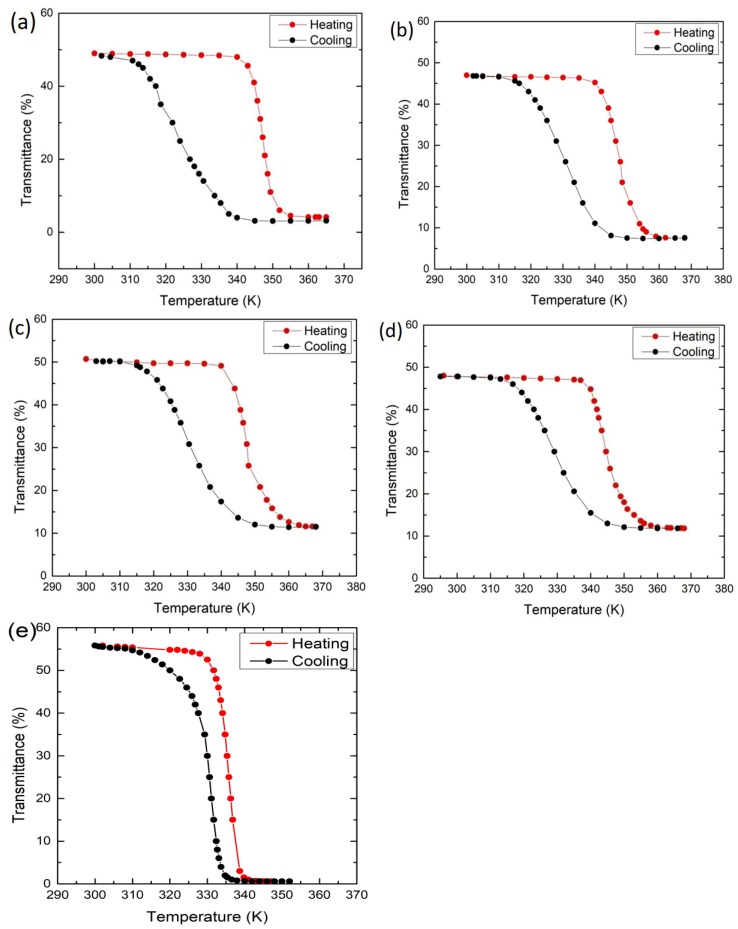
Optical transmittance of the as-deposited VO_2_ thin films under heating (red) and cooling (black): (**a**) 89 V; (**b**) 126 V; (**c**) 146 V; (**d**) 173 V; (**e**) no bias (data extracted from reference [23] and replotted).

**Figure 8:**
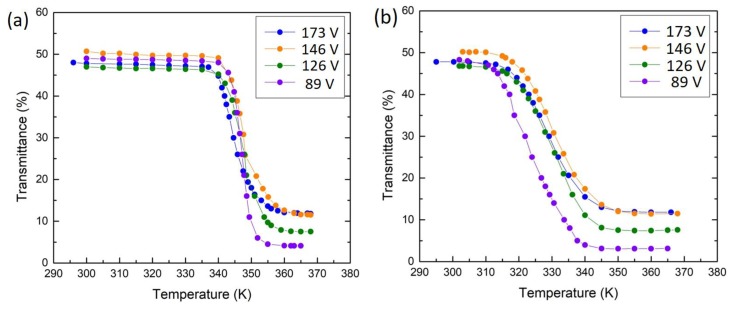
Optical transmittance of the as-deposited VO_2_ thin films with biasing: (**a**) under heating and (**b**) under cooling.

**Figure 9:**
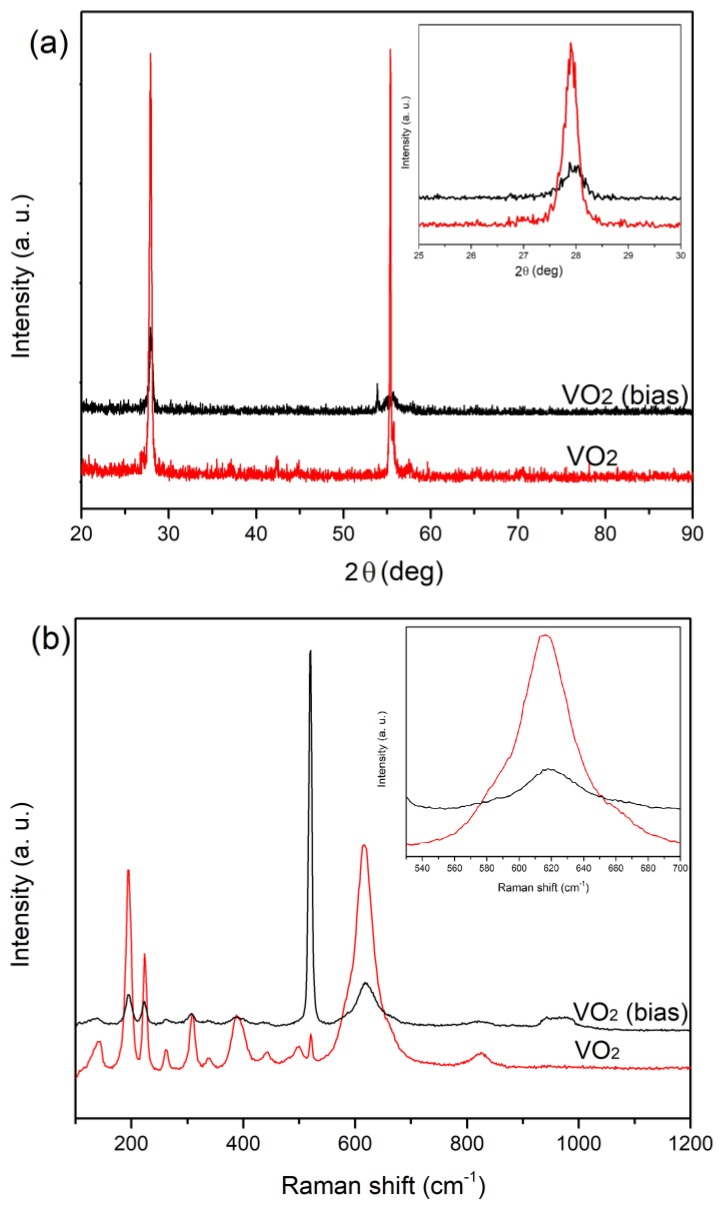
(**a**) X-ray diffraction patterns and (**b**) Raman spectra of VO_2_ thin films (red) and VO_2_ thin films deposited with 173 V bias (black).

Thermal hysteresis loops of transmittance vs temperature are shown in Figure 7. It can be seen that the VO_2_ thin films deposited with a substrate bias voltage from 126 V to 173 V show similar hysteresis with a temperature width (Δ*H*) of 13 °C, while the sample deposited with 89 V bias shows a wider hysteresis with a width of 20 °C. Figure 8 provides a summary of transmittance vs temperature curves under heating and cooling schedules for all the samples; the results are generally consistent with those in Figure 4 and Figure 5 and the implications in 6. The comparison of the hysteresis width (Δ*H*) of 5 °C in VO_2_ thin films deposited without any bias [23], shown in Figure 7e, indicates that the VO_2_ thin films deposited with biasing exhibit quite wider hysteresis loops. The Δ*H* widening can be attributed to grain boundaries and defects induced by the bias, and the enlarged grain size. On the other hand, the transition temperature *T*_t_ of VO_2_ thin films deposited with bias increases significantly compared with those deposited without biasing. VO_2_ thin films deposited without biasing show *T*_t_ of 335 K as we reported previously [23], whereas *T*_t_ of VO_2_ thin films deposited with biasing reaches up to 349 K in this work. Usually the variation of the transition temperature has been associated with the stress and microstructure of the samples [28]. In this work, it is likely that the increased defect density may also lead to an increase of *T*_t_ along the lines in [29]. Another factor is the grain size. The increase of the grain size usually leads to an increase in *T*_t_ and also the width of hysteresis Δ*H* as reported in [9], which is in accordance with our results. Further, the enhanced ion bombardment by biasing often generates residual stress in the as-deposited films [30]. In Figure 9, both the XRD and Raman results of VO_2_ thin film deposited with biasing shows a minor shift towards higher angles or wave numbers compared with a VO_2_ thin film deposited with no bias, indicating the existence of compressive stress which is induced by biasing during deposition. The significant residual stress in the films may make the phase transition harder, thus stabilizing the semiconductor phase. Therefore, the significant *T*_t_ increase (14 K) in this work could be attributed to the combined effect of enlarged grain size, increased defect density, and the residual stress induced by substrate biasing.

## 4. Discussion

The modification of *T*_t_ of VO_2_ thin films have been studied previously by others towards reducing *T*_t_. However studies on increasing *T*_t_ of VO_2_ have been only limited. In this work, the substrate biasing has been shown to be effective in increasing the *T*_t_ of VO_2_. In future works, it would be useful to examine the effects of doping and strain control. 

The thickness dependence of the transmittance in the metal phase in Figure 6 is very clear and leads to an attenuation coefficient of *α* ≈ 3.1 × 10^7^ m^−1^. This high attenuation coefficient represents the absorption of light and can be attributed to free carrier absorption (the Drude model) [31]

(1) $$\alpha \approx \frac{\sigma_{o}}{cn\varepsilon_{o}{\left(\omega \tau \right)}^{2}}$$

where *σ_o_* is the real part of the AC conductivity (taken as the DC conductivity), *c* is the speed of light, *n* is the refractive index, *ε_o_* is the absolute permittivity, *ω* is the angular frequency (2*πc*/*λ*), and *τ* is the mean scattering time of free carriers. If *μ_d_* is the drift mobility, then *μ_d_* = *eτ*/m_e_*, where *e* is the elementary charge and m_e_* is the effective mass of the carriers. The measurement of the conductivity in the present case was rendered difficult due to the semiconducting silicon substrate, but typical values in the literature are 3 × 10^4^ Ω^−1^·m^−1^ for polycrystalline films [32] and 3.8 × 10^5^ Ω^−1^·m^−1^ for epitaxial films [6]. We can take a typical value of 3 × 10^4^ Ω^−1^·m^−1^ for polycrystalline films [32], as in this work, but still need *τ*. The drift mobility in the metallic phase is roughly 0.1 cm^2^·V^−1^·s^−1^ [6] and using an effective mass of 7.1*m_e_*, [33], we can estimate *τ* to be very roughly 4.0 × 10^−16^ s, so that with *n* ≈ 2.2 in the metal phase [34], we find the expected *α* to be very approximately 3.5 × 10^7^ m^−1^. This is very close to the measured value, even though there were four parameters *σ_o_*, *μ_d_*, m_e_*, and *n* used from the literature. If one used an m_e_* = 3.5*m_e_* [35] instead, for example, then *α* is about 14 × 10^7^ m^−1^, even higher. Clearly, free carrier absorption can easily account for the observed attenuation coefficient. We can also evaluate the experimental extinction coefficient *K* at 2000 nm from *α* = 2(2*π*/*λ*)*K*, which gives *K* ≈ 4.9.

The measured transmittance of the VO_2_/Si structure in the insulating phase is obviously influenced by the substrate as well as interference of light within the film. As shown in Figure 4e, for wavelengths greater than about ~1150 nm, the Si substrate alone is fully transparent with *T* ≈ 52.5% with a transmittance that can be shown to be determined by the transmittance through the well-known thick plate equation (with Fresnel reflections) with *n* ≈ 3.50 (e.g., [36]). The transmittance of the VO_2_/Si structure, on the other hand, over the same wavelength range (*λ* > 1150 nm) starts with approximately 30% and rises to about 50% as the wavelength increases from 1150 to 2500 nm. Experiments therefore point to an increase in the attenuation in the VO_2_ insulating phase with decreasing wavelength, or increasing photon energy *hν*. We can explain the change in the transmittance *T* from around 1200 nm to 2500 nm by considering the transmittance of a thin film on a thick transparent substrate. Over the wavelength region where the extinction coefficient is much less than the refractive index, *T* can be written in terms of the Swanepoel equation [37]:

(2) $$T=\frac{Ax}{B-Cx\cos\phi +Dx^{2}}$$

where $A=16n^{2}s$, $B={\left(n+1\right)}^{3}(n+s^{2})$, $C=2(n^{2}-1)(n^{2}-s^{2})$, $D={\left(n-1\right)}^{3}(n-s^{2})$, ϕ=4πnL/λ, x=exp(−αL) in which *s* is the refractive index of the substrate and *α* is the attenuation coefficient in the bulk of the film. We can write *α* as a sum of absorption and scattering contributions as

(3) $$\alpha =\frac{A_{a}}{h\nu }{\left(h\nu -E_{g}\right)}^{1/m}+\frac{A_{s}}{\lambda^{p}}$$

where *A_a_* a constant, *E_g_* is the optical bandgap, and *m* = 2 for direct and *m* = ½ for indirect optical transition, *hν* is the photon energy (*ν* = *c/λ*), *A_s_* is a constant, and *p* is an index that depends on the nature of scattering (e.g., *p* = 4 for Rayleigh scattering). Equations (2) and (3) were fitted to the data with various choices of parameters. 

**Figure 10:**
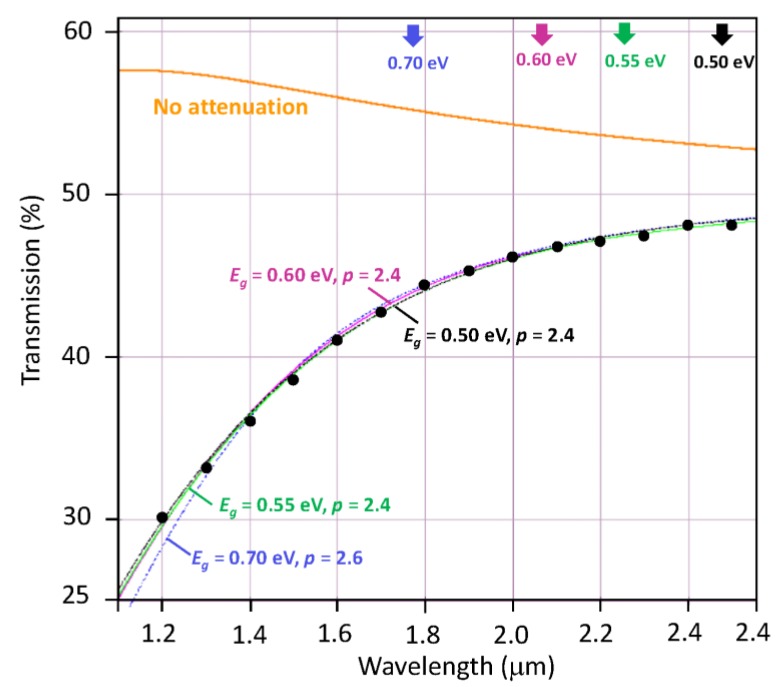
The transmittance vs wavelength for the 102 nm VO_2_ thin film with various contributions. The orange curve is the Swanepoel transmittance with no attenuation. Other colored curves are selected fits to the data as follows: blue: *E_g_* = 0.70 eV, *A_a_* = 0.0070 nm^−1^·eV^−1^, *p* = 2.6, *A_s_* = 5.9 × 10^5^ nm^1.6^**;** magenta: *E_g_* = 0.60 eV, *A_a_* = 0.0048 nm^−1^·eV^−1^, *p* = 2.4, *A_s_* = 1.3 × 10^5^ nm^1.4^**;** green: *E_g_* = 0.55 eV, *A_a_* = 0.0038 nm^−1^·eV^−1^, *p* = 2.4, *A_s_* = 1.30 × 10^5^ nm^1.4^**;** black: *E_g_* = 0.50 eV, *A_a_* = 0.0035 nm^−1^·eV^−1^, *p* = 2.4, *A_s_* = 1.25 × 10^5^ nm^1.4^**.**

Figure 10 shows the experimental transmittance vs wavelength behavior from 1200 nm to 2500 nm. The colored lines are *T* values based on Equations (2) and (3) with *n* ≈ 2.8 [38,39] and *s* = 3.5. The expected thin film transmittance with no attenuation (*α* = 0) cannot explain the data, and neither can just purely band-to-band absorption with typical bandgaps of *E_g_* = 0.6 eV [40,41] and 0.7 eV [6,42] with direct and indirect transitions (not shown), which means that there must be some scattering of light in the film and/or on the surfaces to account for the observed *T* vs *λ* behavior. Theoretical predictions based on Equations (2) and (3) with *E_g_* = 0.5 eV to 0.7 eV and *m* = ½ are shown in Figure 10 for the best-fit values of *A_a_*, *A_s_*, and *p* given in the figure caption. While *E_g_* = 0.6 eV appears as a perfect fit, *E_g_* = 0.55 eV is also very close. Direct transitions (*m* = 2) could not be fitted at all. Thus, we can conclude that the observed transmittance is in agreement with an indirect bandgap of 0.55–0.60 eV. The scattering index *p* is 2.4 but it should be remembered that any surface scattering in the present analysis based on Equations (2) and (3) would artificially inflate the scattering term and modify*p*. Similar good fits were found for the other samples (not shown) with values that are close to those of sample A. Table 2 summarizes the best-fit parameters for *p* and *As* for all the films with *E_g_* = 0.60 eV and *A_a_* kept the same for all samples; the same absorption coefficient. *A_s_L* represents the total effective scattering in the film. No unambiguous correlation could be established with grain size inasmuch as fitting a multivariable function as in the present case will invariably have uncertainties. Nonetheless, we can conclude that the insulating phase possesses an indirect bandgap of 0.60 eV ± 0.5 eV, and the films exhibit significant scattering of light.

Finally, we can check the validity of the above arguments by noting that the strongest attenuation occurs at 1200 nm, where, using the best-fit values, the highest extinction coefficient (*K*) is 0.57. This is still smaller than *n* = 2.8 by a factor of 5, which provides support for the use of Equation (2) [37].

## 5. Conclusions

The present work clearly shows that applying substrate biasing can modify the microstructure and optical properties of VO_2_ thin films deposited by magnetron sputtering. By applying substrate biasing, the deposition rate of the as-deposited VO_2_ thin films decreases while the grain size increases. The films prepared under a higher bias voltage are thinner and hence show higher transmission in the metallic state. The defects induced by the substrate biasing degrade the optical properties of VO_2_ in the sense that the hysteresis temperature width Δ*H* of the transition temperature observed during heating and cooling becomes poor. Applying substrate biasing also alters the characteristics of the phase transition. The transition temperature of VO_2_ thin films deposited under bias increases 14 K accompanied by a widened hysteresis curve. 

An advantage of the increased transition temperature of VO_2_ thin films is the enhancement of thermal stability of VO_2_ thin films for various electronic and optoelectronic device applications utilizing optical switches, transistors, sensors, and actuators, in which the working temperatures are possibly higher than 68 °C due to the accumulated heat during service.

## Figures and Tables

**Table 1:** Grain size and thickness of the as-deposited VO_2_ thin films with substrate biasing.

Sample #	Biasing Power (W)	Biasing Voltage (V)	Average Grain Size (nm)	Thickness (nm)
A	15	89	58.8 ± 0.6	102 ± 1
B	25	126	67.2 ± 1.0	93 ± 2
C	35	146	88.6 ± 1.2	68 ± 1
D	45	173	75.1 ± 0.5	60 ± 2

**Table 2:** The scattering contribution to attenuation in VO_2_ films on Si, given *E_g_* = 0.60 eV and *A_a_* = 0.0048 nm^−1^·eV^−1^.

Sample #	Thickness (nm)	Average Grain Size (nm)	*p*	*A*_s_ (nm*^p^*^−1^)	*α_s_L* at 2000 nm
A	102 ± 1	58.8 ± 0.6	2.4	1.30 × 10^5^	0.16
B	93 ± 2	67.2 ± 1.0	1.6	4.05 × 10^2^	0.20
C	68 ± 1	88.6 ± 1.2	2.0	6.70 × 10^3^	0.11
D	60 ± 2	75.1 ± 0.5	2.2	3.65 × 10^4^	0.12
