# Erratum: Zhang, C. et al., The Effect of Substrate Biasing during DC Magnetron Sputtering on the Quality of VO_2_ Thin Films and Their Insulator–Metal Transition Behavior. *Materials* 2019, *12*, 2160

**DOI:** 10.3390/ma13225132

**Published:** 2020-11-13

**Authors:** Chunzi Zhang, Ozan Gunes, Yuanshi Li, Xiaoyu Cui, Masoud Mohammadtaheri, Shi-Jie Wen, Rick Wong, Qiaoqin Yang, Safa Kasap

**Affiliations:** 1 Department of Electrical and Computer Engineering, University of Saskatchewan, 57 Campus Drive, Saskatoon, SK S7N 5A9, Canada; chz616@mail.usask.ca (C.Z.); ozg372@mail.usask.ca (O.G.); 2 Department of Mechanical Engineering, University of Saskatchewan, 57 Campus Drive, Saskatoon, SK S7N 5A9, Canada; yuanshi_li@yahoo.com (Y.L.); msctaheri@gmail.com (M.M.); qiaoqin.yang@usask.ca (Q.Y.); 3 Canadian Light Source Inc., 44 Innovation Boulevard, Saskatoon, SK S7N 2V3, Canada; xiaoyucls@gmail.com; 4 Cisco Systems Inc., San Jose, CA 95134, USA; shwen@cisco.com (S.-J.W.); rickwon@cisco.com (R.W.)

The authors would like to correct a typographical error in their paper [1]. The abstract and page 11 states 0.60 eV ± 0.5 eV for the bandgap. This should be 0.60 eV ± 0.05 eV, as apparent in the analysis and discussions in Section 4. 
